# Improvements in the order, isotropy and electron density of glypican-1 crystals by controlled dehydration

**DOI:** 10.1107/S0907444913025250

**Published:** 2013-11-19

**Authors:** Wael Awad, Gabriel Svensson Birkedal, Marjolein M. G. M. Thunnissen, Katrin Mani, Derek T. Logan

**Affiliations:** a Department of Biochemistry and Structural Biology, Centre for Molecular Protein Science, Lund University, Box 124, 221 00 Lund, Sweden; b Department of Biophysics, Faculty of Science, Cairo University, Cairo, Egypt; c Department of Experimental Medical Science, Division of Neuroscience, Glycobiology Group, Lund University, Biomedical Center A13, 221 84 Lund, Sweden; d MAX IV Laboratory, Lund University, Box 188, 221 00 Lund, Sweden

**Keywords:** glypican-1, crystal dehydration, HC1, optimization, diffraction anisotropy, crystal packing

## Abstract

The use of controlled dehydration for improvement of protein crystal diffraction quality is increasing in popularity, although there are still relatively few documented examples of success. A study has been carried out to establish whether controlled dehydration could be used to improve the anisotropy of crystals of the core protein of the human proteoglycan glypican-1. Crystals were subjected to controlled dehydration using the HC1 device. The optimal protocol for dehydration was developed by careful investigation of the following parameters: dehydration rate, final relative humidity and total incubation time *T*_inc_. Of these, the most important was shown to be *T*_inc_. After dehydration using the optimal protocol the crystals showed significantly reduced anisotropy and improved electron density, allowing the building of previously dis­ordered parts of the structure.

## Abstract 2

The anisotropy of crystals of glypican-1 was significantly reduced by controlled dehydration using the HC1 device, allowing the building of previously disordered parts of the structure.

## 1. Introduction   

Glypicans are heparan sulfate proteoglycans that are attached to the cell-membrane surface by glycosylphosphatidylinositol anchorage. Glypican-1 (Gpc-1) is the predominant heparan sulfate proteoglycan in the developing and adult human brain and is involved in regulation of neurogenesis, axon guidance and synaptogenesis (Filmus *et al.*, 2008 ▶; Fransson, 2003 ▶; Jen *et al.*, 2009 ▶; Fico *et al.*, 2011 ▶; Dwivedi *et al.*, 2013 ▶). Recently, we determined the crystal structure of N-glycosylated human glypican-1 core protein at 2.55 Å resolution, which revealed a cylindrical all-α-helical fold (dimensions 120 × 30 × 30 Å) decorated with three major loops and containing the 14 cysteine residues that are conserved in all members of the glypican family (Svensson *et al.*, 2012 ▶). The Gpc-1 crystals were delicate, highly fragile plates with typical dimensions of around 0.8 × 0.3 × 0.05 mm which displayed poor isomorphism, with a solvent content of 54%. Gpc-1 crystallizes in space group *P*2_1_, with typical unit-cell parameters *a* = 47.2, *b* = 168.7, *c* = 147.8 Å, β = 94.6°. Variation in unit-cell parameters was noticed between different crystals; in particular, the length of the *c* axis varied between 147 and 154 Å. These crystals also diffracted anisotropically, as reflected in a Wilson *B* factor that was twice as large in the c* direction as in the a* and b* directions, which limited the effective resolution to 2.9 Å in the c* direction. Refinement against ellipsoidally truncated data produced by the UCLA MBI Diffraction Anisotropy Server (http://services.mbi.ucla.edu/anisoscale) did not improve the density map, so we used the original anisotropic 2.55 Å resolution data in the refinement of the structure deposited with PDB entry 4acr (Svensson *et al.*, 2012 ▶).

The anisotropy observed in Gpc-1 crystals is largely a result of intrinsic disorder in the lattice packing, where there are more lattice contacts in the *a* and *b* directions than in the *c* direction. The asymmetric unit of Gpc-1 crystals, in space group *P*2_1_, contains four chains in two pairs, *A*/*B* and *C*/*D*, related by translational pseudosymmetry. In the *a* and *b* directions strongly packed layers are formed through heterotypic contacts between the chains in the *A*/*B* and *C*/*D* pairs and homotypic interactions between identical chains in adjacent cells related by translation along the *a* axis (total area of 2121 Å^2^). In contrast, the layers are held together by a single interaction in the *c* direction between chain *B* and chain *C* related by a pure *x* = 1 translation (one unit cell along the *a* axis), the buried surface area of which is only 275 Å^2^. Different strategies to overcome such diffraction-quality problems have been described in the literature, including the use of post-crystallization treatments such as crystal annealing, tempering, soaking, chemical cross-linking and dehydration (Newman, 2006 ▶; Heras & Martin, 2005 ▶). Annealing, cross-linking and chemical dehydration of Gpc-1 crystals did not produce reproducible diffraction improvements, but controlled dehydration was successful in our hands, as shown in the present study. Clearly, dehydration is potentially of great interest for crystals with high solvent content and/or poor order, where reduction of the solvent content might yield crucial improvements in the crystal packing and consequent improvements in the diffraction quality (Heras *et al.*, 2003 ▶; Heras & Martin, 2005 ▶). Lower symmetry space groups (triclinic, monoclinic and orthorhombic) have a greater success rate for crystal improvement by dehydration, as there are fewer restrictions on the transitions of the molecules (Sanchez-Weatherby *et al.*, 2009 ▶). Many cases of diffraction improvement of protein crystals by dehydration have been surveyed by Russo Krauss *et al.* (2012 ▶).

Various protocols have been developed for protein crystal dehydration, including air dehydration, the addition of a dehydrating solution to the crystallization drop and soaking with dehydrating compounds (Heras *et al.*, 2003 ▶; Newman, 2006 ▶; Heras & Martin, 2005 ▶). All of these methods are crystal- and time-consuming, and the outcome is essentially unknown until the experiment is finished. In order to dehydrate crystals more reproducibly, a number of devices have been designed to control the relative humidity (RH) surrounding the crystal. One of the first successful attempts to automate and control the process was the free-mounting system (FMS), which is more effective and reproducible than using chemical methods (Kiefersauer *et al.*, 1996 ▶, 2000 ▶). A stream of humid air produced by mixing two air streams of 0 and 100% RH is applied to control the humidity around the crystal. Despite the successful diffraction improvement made by FMS in many cases (Kiefersauer *et al.*, 2000 ▶; Kyrieleis *et al.*, 2005 ▶; Bowler *et al.*, 2006 ▶; Hagelueken *et al.*, 2012 ▶), the system requires careful handling and is not compatible with the crowded sample environment at a synchrotron.

More recently, a humidity-controlled device (HC1) was designed to be better suited for use at synchrotrons with no disruption to the experimental environment (Sanchez-Weatherby *et al.*, 2009 ▶). The HC1 delivers a controlled humidified air stream (*via* its air-dispensing nozzle) of a precise RH that can be used to alter the solvent content inside the macromolecular crystals. Samples are mounted in standard cryo-loops. The progress of the dehydration can be followed by the HC1 control software, which monitors and controls the humidity changes and displays a live image of the crystal. Control of the RH can be programmed either as a gradient, stepwise or a combination of both. Different variables of the dehydration protocol usually need to be optimized to induce the most ordered and reproducible rearrangement of the molecules in the crystal. Such variables include the RH step size, the dehydration rate, the equilibration time, annealing, the number of steps and the total time for the protocol. Once the optimum hydration level is obtained, cryocooling of the treated crystals is easy to perform by hand or using a sample-changer robot. The components and operation of HC1 and some experimental methods have been described in detail with some examples (Sanchez-Weatherby *et al.*, 2009 ▶; Russi *et al.*, 2011 ▶). The ease and simplicity of HC1 operation make the dehydration experiment achievable within a reasonable time.

There are relatively few documented examples of successful application of the HC1 and we were interested to determine whether it could improve diffraction quality in our anisotropic Gpc-1 crystals. Another goal was to investigate whether we could increase the crystal symmetry from monoclinic to orthorhombic, which we thought possible owing to the near-orthorhombic unit-cell parameters (β ≃ 94°) and a pseudo-translation vector in the native Patterson. Experimental approaches for optimizing the dehydration protocol for Gpc-1 crystals were investigated. Clear improvements in the quality of the diffraction were obtained, enabling the building of segments of the model that were previously disordered.

## 2. Methods   

### 2.1. Protein expression, purification and crystallization   

C-terminally truncated His-tagged Gpc-1 protein lacking the endogenous signal peptides and the heparan sulfate attachment domain (residues 24–479; UniProt identifier P35052) was expressed in stable HEK293 cells as described previously (Svensson *et al.*, 2012 ▶). The conditioned medium was collected and the protein was purified from it by nickel–NTA affinity chromatography. The purified protein was dialyzed into 20 m*M* Tris pH 8 and was concentrated by ultrafiltration. The protein concentration was measured using a NanoDrop spectrophotometer (NanoDrop, Wilmington, Delaware, USA). Crystallization was performed using sitting-drop vapour diffusion by mixing 2 µl 15 mg ml^−1^ Gpc-1 protein with 2 µl reservoir solution consisting of 11–13% PEG 6000, 0.1 *M* Tris–HCl pH 8, 0.2 *M* CaCl_2_ and equilibrating over 0.5 ml reservoir solution. Large thin plate-like crystals with dimensions of around 0.8 × 0.3 × 0.05 mm grew within a week.

### 2.2. Crystal dehydration   

Saturated ammonium sulfate and sodium chloride salt solutions were used to calibrate the HC1 machine installed online at station I911-3 of the MAX IV Laboratory, Lund, Sweden (Ursby *et al.*, 2013 ▶) as described previously (Sanchez-Weatherby *et al.*, 2009 ▶). The approximate RH of the Gpc-1 cryosolution (13% PEG 6000, 0.2 *M* CaCl_2_, 0.1 *M* Tris pH 8.0, 15% ethylene glycol) was found by running the double-gradient script to adjust the RH from 99 to 90% RH and back again while monitoring the size of the drop using the HC1 software. The RH of the humid air was modified until it was in equilibrium with the drop. The drop remained the same size between 95 and 96% RH and this was thus used as the initial RH (RH_i_) for all further experiments.

Gpc-1 crystals were mounted on mesh LithoLoops (Molecular Dimensions, Newmarket, England) of sizes 0.2 and 0.3 mm, briefly soaked in the cryosolution and finally mounted in the HC1 air stream at RH_i_ = 95% for dehydration. Excess liquid was removed from the opposite side of the mesh loop to the crystal using a paper wick. An initial diffraction image was collected at room temperature to judge the crystal quality. The smallest available beam diameter of 30 µm combined with short X-ray exposures (about 5–10 s per image) was used to expose the crystal minimally and limit radiation damage yet still permit successful indexing. Lattice parameters and relative diffraction resolution were monitored using *iMosflm* (Battye *et al.*, 2011 ▶) to assess lattice changes. Six to ten images were typically collected at room temperature from each Gpc-1 crystal at different relative humidities, translating the crystal by more than 50 µm between exposures.

The variations in the dehydration protocols for each experiment are described in §[Sec sec3]3. Briefly, a set of parameters was tested, including the final RH (RH_f_; from 85 to 95%), the dehydration rate (per 0.1% RH; 10–90 s) and lastly the total incubation time *T*_inc_ of the crystal in the humid air stream, including all dehydration and equilibration, before crystal cooling. After dehydration (without exposure to X-rays), the crystal was unmounted into an empty vial containing liquid N_2_ using the CATS sample changer (IRELEC, Saint-Martin-d’Hères, France) and stored in liquid N_2_ for subsequent diffraction testing. Once a sufficient number of crystals had been harvested, the Cryostream (Oxford Cryosystems, Oxford, England) was remounted and diffraction data were collected from each crystal at 100 K.

### 2.3. Data collection and computational analysis   

X-ray diffraction data were collected from cooled dehydrated crystals at 100 K on station I911-3. Diffraction images were indexed, integrated and scaled using *XDS* (Kabsch, 2010 ▶) and were further processed using programs from the *CCP*4 (Winn *et al.*, 2011 ▶) and *PHENIX* (Adams *et al.*, 2010 ▶) packages. Analysis of X-ray data sets was performed using *phenix.xtriage* and *SFCHECK* (Vaguine *et al.*, 1999 ▶). The structures were solved using *AutoMR* in *PHENIX* with *Phaser* (McCoy *et al.*, 2007 ▶) against a dimer consisting of chains *C* and *D* of PDB entry 4acr as a starting model. The initial models were completed by manual building in *Coot* (Emsley *et al.*, 2010 ▶) followed by rounds of refinement using *REFMAC*5 (Murshudov *et al.*, 2011 ▶) and, in the final stages, *phenix.refine* (Afonine *et al.*, 2012 ▶). The models were validated using *MolProbity* (Chen *et al.*, 2010 ▶). Graphical representations were generated using *Coot* and the *PyMOL* Molecular Graphics System (v.1.5; Schrödinger, New York, USA). Structural alignments were created in *PyMOL* *via* an initial sequence alignment. Crystal packing and total interface area were evaluated by *PISA* analysis (Krissinel & Henrick, 2007 ▶). The coordinates and the diffraction data of the glypican-1 structure from crystals dehydrated to 86% using the optimal protocol have been deposited in the Protein Data Bank with accession code 4bwe.

## 3. Results and discussion   

Gpc-1 crystallizes in space group *P*2_1_, with typical unit-cell parameters *a* = 47.2, *b* = 168.7, *c* = 147.8 Å, β = 94.6° (Svensson *et al.*, 2012 ▶). These crystals have a solvent content of 54%. Before dehydration the *c* parameter typically varied from 147 to 154 Å for cryocooled crystals. The Gpc-1 crystals diffracted to 2.55 Å resolution, albeit with significantly higher diffraction intensity falloff with resolution along the c* direction than along a* and b*, which indicates anisotropy of the data. The weak reflections along c* mostly contain noise and thus generate map noise, while the detailed information carried by high-resolution reflections (along a* and b*) is suppressed (Rupp, 2010 ▶). This anisotropy produced poor quality density maps that were particularly lacking in detail for some of the extremities of the molecule. The Gpc-1 data were analysed using the UCLA MBI Diffraction Anisotropy Server (Strong *et al.*, 2006 ▶), which uses *Phaser* to calculate the anisotropy (McCoy *et al.*, 2007 ▶). This analysis revealed that the *B* factor in the c* direction was 40.7 Å^2^ higher than in the a* and b* directions, confirming the strong anisotropy. The server recommended resolution limits of 2.55, 2.55 and 2.90 Å along the a*, b* and c* directions, respectively. We refined the model against an ellipsoidally truncated data set to the resolution limits provided by the UCLA MBI Diffraction Anisotropy Server, but no improvements in the density maps or the model were achieved.

As noted, some parts of the structure (PDB entry 4acr) were partially disordered and were not visible in the initial electron-density map. Also, there was a variation in map quality between the different monomers (no noncrystallographic symmetry restraints were used in the refinement). Finally, the average *B* factor of 4acr was quite high (73.8 Å^2^) when compared with the *B* factors of 706 PDB entries of similar resolution (which ranged from 8.3 to 68 Å^2^ with a mean value of 40.7 Å^2^) using *phenix.polygon* (Urzhumtseva *et al.*, 2009 ▶).

### 3.1. Initial characterization of the dehydration effect   

**Figure 1:**
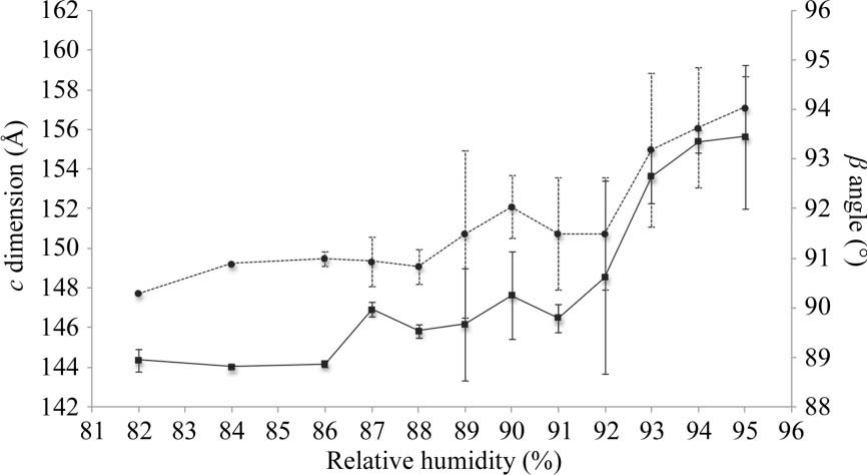
Tracking the effect of controlled dehydration on the unit-cell parameters *c* (solid line) and β (dotted line) of monoclinic glypican-1 crystals. The plotted values are averaged from two separate experiments; the standard deviation is shown as an error bar. All data were collected at room temperature (∼298 K). Crystals were dehydrated from 95 to 80% RH at 0.1% RH per minute.

The initial dehydration experiments were solely designed to determine whether dehydration causes a change in the crystal packing of Gpc-1 crystals by monitoring the unit-cell parameters. Gpc-1 crystals were mounted in the HC1 device at RH_i_ = 95%. An initial diffraction image was collected from each crystal at room temperature to judge the crystal quality with a minimal exposure time. The RH was reduced in a single gradient in 1% RH steps at 0.1% RH per minute, each step being followed by a short equilibration time (5 min) allowing the crystal to stabilize. Consecutive images were collected from different parts of the crystal that were not affected by radiation damage until a final RH of 80% was reached. The experiment was carried out twice for the whole range between 95 and 80% RH, each time with a fresh crystal. A small circular beam of 30 µm was used in order to maximize the number of data points per crystal. Thus, each dehydration series was performed on the same crystal, which was possible because of their large size in two dimensions. The unit-cell parameter most sensitive to dehydration was the *c* axis, which shortened from 158 Å at 95% RH to 145 Å at 82% RH (Fig. 1 ▶). This contraction was accompanied by a decrease in the β angle from 94.5 to 90.3° (Fig. 1 ▶). In contrast, the *b* axis decreased by less than 2 Å and no change was observed for the *a* axis (not shown). An increase in the resolution of the diffraction pattern was observed using *iMosflm* at values down to 86–88% RH, but the diffraction pattern deteriorated with further dehydration and the crystals had lost all diffraction by 80% RH. The observed large error bars in the unit-cell parameters in the area between 89 and 95% RH (Fig. 1 ▶) could have several origins: (i) errors in the measurements, since the unit-cell parameters were calculated by *iMosflm* using one image, which is not always sufficient for cell refinement, (ii) the crystals might be undergoing a phase transition that produces an instability in the unit-cell volumes within this range of RH or (iii) the starting unit-cell volumes at 95% RH typically vary from crystal to crystal and thus their initial shrinkage response could also vary. Of these scenarios, (i) is possibly less likely, since the crystal was in the same orientation for each exposure. In any case, the error bar is significantly smaller after 89% RH, which means that dehydration succeeded in stabilizing the unit-cell volume in correlation with the RH after that value.

Many crystals were tested for dehydration and all responded by a shrinkage in the unit-cell volume that was reflected in the crystal packing and the model quality. Thus, glypican crystals undergo a transition upon controlled dehydration. Whether this transition has a beneficial or a detrimental effect on the diffraction quality may depend on the dehydration protocol. The next step in our investigation was to fine-tune the protocol to yield the best dehydration gain by cooling crystals and collecting full data sets at cryogenic temperature.

### 3.2. Which parameters is it most important to optimize?   

#### 3.2.1. The effect of RH_f_ and dehydration rate   

**Figure 2:**
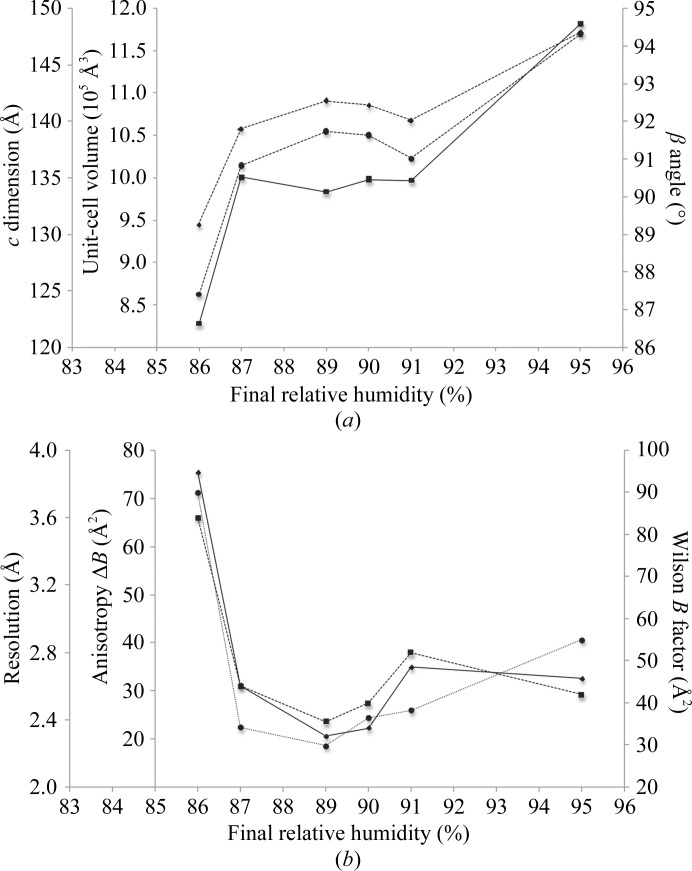
Influence of controlled dehydration to different RH_f_ on (*a*) unit-cell parameters and (*b*) diffraction data quality of Gpc-1 crystals. All crystals were dehydrated at 0.1% RH per minute and flash-cooled; full data were then collected at 100 K. (*a*) The changes in β angle (squares), *c* dimension (circles) and unit-cell volume (diamonds) with RH_f_ are plotted as solid, dotted and dashed lines, respectively. (*b*) The diffraction data quality as estimated by resolution (squares), anisotropy Δ*B* (circles) and Wilson *B* factor (diamonds) is plotted as solid, dotted and dashed lines, respectively. The resolution limit is defined as the resolution where the mean *I*/σ(*I*) is approximately 2.0.

A number of crystals were dehydrated to different RH_f_ values between 96 and 86% using the single-gradient script (RH_f_, 0.1%, 60 s), which means dehydration to a certain RH (RH_f_) by lowering the RH in 0.1% steps with 60 s per step. The crystals were then equilibrated at RH_f_ for 15 min, harvested by the CATS robot and cryocooled. Subsequently, full data sets were collected at 100 K, which manifested the reduction in the *c* axis, the β angle and the overall unit-cell volume with dehydration and showed them to be reproducible between 91 and 87% RH_f_ (Fig. 2 ▶ *a*). To evaluate the dehydrated crystal quality, the change in diffraction resolution limit, the Wilson *B* factor and the anisotropy Δ*B* are plotted *versus* the RH_f_ (Fig. 2 ▶ *b*). All diffraction properties improved with a reduction in the unit-cell volume, particularly between an RH_f_ of 87 and 90%. However, with additional dehydration beyond 87% RH the Wilson *B* factor and anisotropy increased and the resolution worsened. The best data, to 2.5 Å resolution with a mild anisotropy Δ*B* of 18.7 Å^2^, were collected at an RH_f_ of 89% and not at 86%, as concluded from the room-temperature experiment. This suggested that the outcome of dehydration might partially depend on other parameters of the protocol such as the dehydration rate.

**Figure 3:**
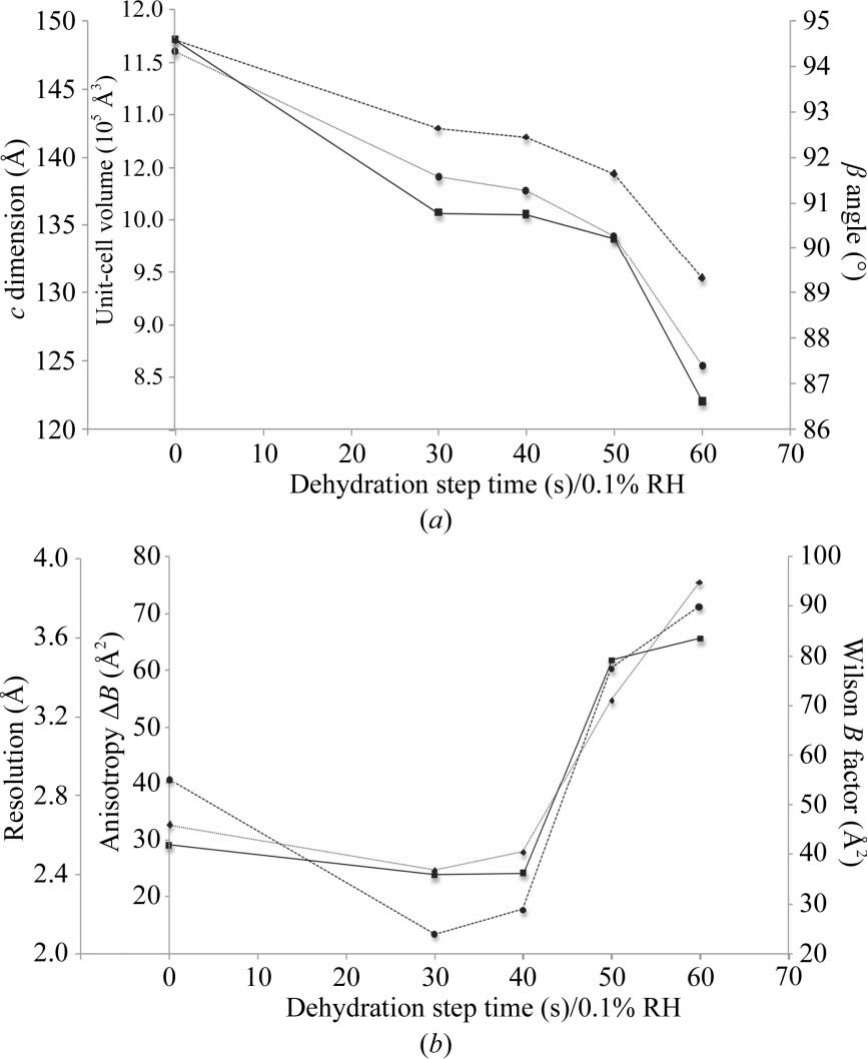
Effect of controlled dehydration to RH_f_ = 86% at different rates on (*a*) the unit-cell parameters and unit-cell volume and (*b*) the diffraction data quality of Gpc-1 crystals. The data at zero dehydration rate were for a control non-dehydrated crystal. All other crystals were dehydrated to RH_f_ = 86% in 0.1% steps with the given step time and flash-cooled; full data sets were then collected at 100 K. (*a*) The changes in β angle (squares), *c* dimension (circles) and unit-cell volume (diamonds) with different dehydration rates are plotted as solid, dotted and dashed lines, respectively. (*b*) The diffraction data quality as estimated by resolution (squares), anisotropy Δ*B* (circles) and Wilson *B* factor (diamonds) is plotted as solid, dotted and dashed lines, respectively.

To further investigate the role of the dehydration rate, numerous Gpc-1 crystals were dehydrated to an RH_f_ of 86% at different rates (0.1% steps for 30, 40, 50 or 60 s) followed by an equilibration time of 15 min and then harvesting and cryocooling and collection of full data sets. If the Gpc-1 crystal was slowly dehydrated, the unit cell shrank (Fig. 3 ▶ *a*) but the resolution, *B* factor and anisotropy worsened (Fig. 3 ▶ *b*), which might be owing to the rate itself or to the longer total incubation time. Therefore, the role of the total incubation time of the crystal was further explored.

#### 3.2.2. The effect of total incubation time (*T*_inc_) on crystal packing and diffraction quality   

**Figure 4:**
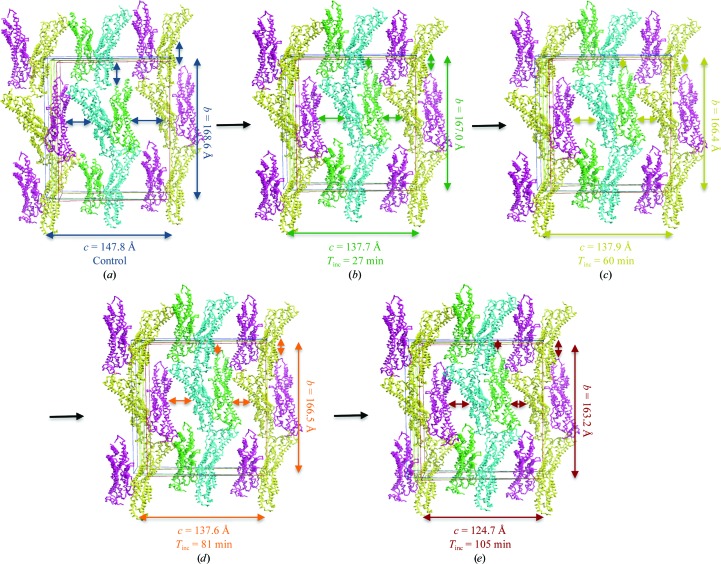
Gpc-1 crystal packing in selected dehydration states. The view is along the unit-cell *a* axis. The asymmetric unit and symmetry-related molecules are shown as C^α^ traces coloured by chain (*A*, green; *B*, blue; *C*, red; *D*, yellow). The unit-cell boxes, arrows and *b* and *c* unit-cell parameters are coloured by dehydration state: (*a*) control (no dehydration), blue; (*b*) 27 min, green; (*c*) 60 min, yellow; (*d*) 81 min, orange; (*e*) 105 min, red. Coloured arrows show the improvement in the intermolecular packing with dehydration.

The dehydration of many crystals was carried out using various RH_f_ and dehydration rates followed by the same equilibration time of 15 min. Table 1 ▶ summarizes the data statistics arranged according to the total incubation time *T*_inc_ of a crystal in the humid air stream before cryocooling it for data collection. As *T*_inc_ increases, the unit-cell volume and solvent content decrease, which results in improved crystal packing (Fig. 4 ▶).

In this set of experiments it appeared that the shrinkage of the unit cell was not dependent on the dehydration rate or the RH_f_ as long as the latter was in the range 86–89%. Analysis of these results revealed that dehydration of Gpc-1 crystals for a *T*_inc_ of between 60 and 81 min resulted in isomorphous unit-cell volumes between crystals and in an increased diffraction quality. Within this range of *T*_inc_ the diffraction resolution systematically improved to ∼2.5 Å with similar acceptable values for the anisotropy Δ*B* and Wilson *B* factor. Several crystals were dehydrated within this time range to different RH_f_ (89, 88, 87 and 86%) using different dehydration rates (0.1% steps for 60, 40 and 30 s) and a reproducible outcome was achieved. In contrast, the diffraction pattern deteriorated if the Gpc-1 crystals were incubated in the machine for longer than ∼81 min and all diffraction was lost if they were incubated for 120 min or more.

Structures were determined from all dehydrated data sets by molecular replacement using *PHENIX*/*Phaser* with the dimer consisting of chains *C* and *D* from PDB entry 4acr as a search model. A few rounds of manual rebuilding in *Coot* and refinement with *phenix.refine* were performed. The structures were compared by explicitly superimposing all C^α^ atoms on their equivalents in the other structures using *PyMOL* (*i.e.* allowing no rejection of outliers). The r.m.s.d. between all aligned structures of crystals dehydrated for 60–80 min was 0.4 Å, *i.e.* they showed very little divergence from each other. Larger differences were observed between respective chains when comparing the dehydrated structure with the control structure 4acr. The pairwise r.m.s.d. values were 1.15, 0.61, 0.70 and 0.60 Å for chains *A*–*D* based on the alignment of 335, 407, 381 and 412 atoms, respectively, using the *SSM* algorithm in *Coot*.

To follow the pathway of crystal-packing improvement, a comparison was made between the molecules inside the unit cell for numerous models obtained from the dehydrated crystals for *T*_inc_ ranging from 0 to 105 min. In the first stage the unit cell starts shrinking in two directions, along the *b* and *c* axes (Figs. 4 ▶ *a* and 4 ▶ *b*); with longer dehydration times the intermolecular interactions increase along the *c* axis, resulting in an isomorphous unit-cell volume over a *T*_inc_ range of 60–80 min, accompanied by similar packing inside the crystal (Figs. 4 ▶ *c* and 4 ▶ *d*). Further dehydration increased the packing along the *c* dimension, but with a detrimental effect on the diffraction quality. This reveals the importance of taking incubation times into account when planning a controlled protein crystal dehydration experiment using the HC1 device.

### 3.3. How does the dehydration enhance diffraction and model quality?   

**Figure 5:**
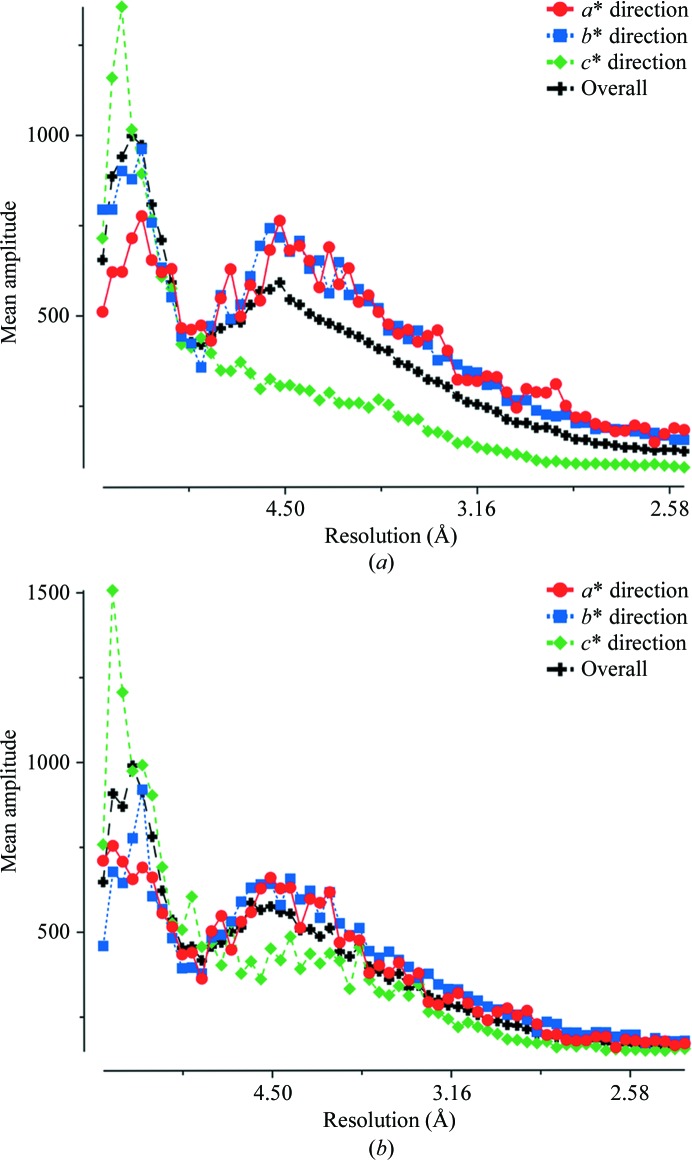
Intensity fall-off plots of (*a*) control and (*b*) dehydrated Gpc-1 data generated by the *TRUNCAT*E program from the *CCP*4 package.

The best diffraction data set (2.46 Å resolution) was collected from a crystal dehydrated using the script (86%, 0.1%, 30 s) with a total incubation time of 65 min (Table 2 ▶). These data had an anisotropy Δ*B* of 13.6 Å^2^ when checked with the UCLA MBI Diffraction Anisotropy Server, while the control data, without dehydration, had a strong anisotropy Δ*B* of 40.7 Å^2^. The resolution fall-off plots of the dehydrated data show little anisotropy (Fig. 5 ▶) and the reciprocal-space pseudo-precession images reflect the improvement in diffraction after dehydration, where more strong reflections are observed along the c* direction in the *h*0*l* and 0*kl* planes (data not shown). The output of *SFCHECK* (Vaguine *et al.*, 1999 ▶) from the *CCP4* package (Winn *et al.*, 2011 ▶) also indicated severe anisotropy in the control data, with eigenvalue ratios of 0.4, 0.4, 1.0 which distinctly deviate from the isotropic value of 1.0. Dehydration resulted in improved eigenvalue ratios of 0.75, 0.71, 1.0, indicating that the crystal becomes more isotropic after dehydration. Also, the Wilson *B* factor was reduced by 25% to 36.8 Å^2^ (Table 2 ▶), which reveals significant improvement in the degree of short-range lattice order in the crystal after dehydration. Because of time constraints during data collection, the dehydrated data set has lower multiplicity than the 4acr data set. One interpretation of the improvements is that they are a consequence of reduced radiation damage in the dehydrated data set owing to lower overall exposure time; however, this is unlikely as all data sets from non-dehydrated crystals, even with lower multiplicity, suffered from the same anisotropy.

**Figure 6:**
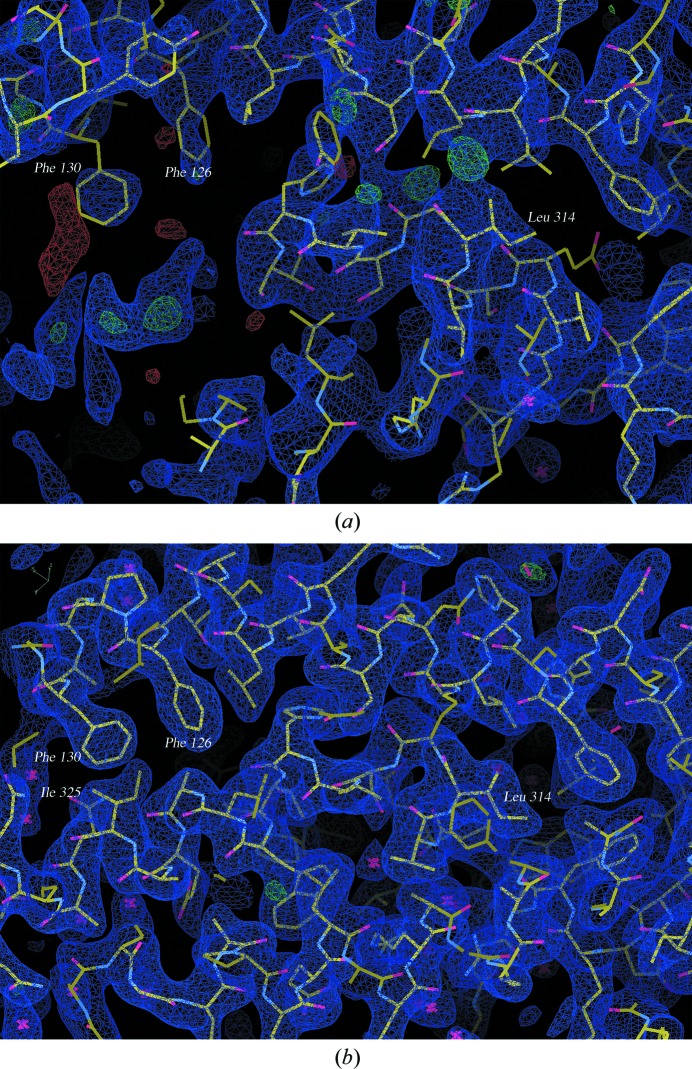
2|*F*_o_| − |*F*_c_| electron-density map contoured at 1σ (blue) in the protease-sensitive region of chain *A*, where significant differences between the control structure (*a*) and the dehydrated structure (*b*) exist. This figure was generated using *Coot*.

Anisotropic diffraction attenuation is the result of an anisotropic distribution of all of the types of displacements such as lattice disorder, variations in molecular conformation, intermolecular motion, local anisotropic atomic displacement or any other displacement effects. Disorder in a protein crystal is frequently anisotropic because adequate intermolecular interactions may exist in only two dimensions or in layers (as is often the case in membrane proteins), while contacts in the third dimension may be weak (Rupp, 2010 ▶). The molecules in the glypican-1 crystals are more tightly packed along the *a* and *b* directions than in the *c* direction. Shrinkage of the unit cell after dehydration leads to tighter packing, decreasing the flexible areas and forming new lattice contacts, mainly along the *c* axis. Concomitantly, the water loss from the crystal by dehydration induces mechanical forces which may rearrange locally disordered areas (Sanchez-Weatherby *et al.*, 2009 ▶). All this leads to marked improvement in the diffraction quality, which generates better, less noisy electron-density maps. The new density maps allowed the building of more complete models for all of the Gpc-1 monomers in the asymmetric unit and displayed well defined side-chain density, allowing more reliable side-chain placement (Fig. 6 ▶). Furthermore, the maps facilitated the location of more water molecules (Table 2 ▶).

**Figure 7:**
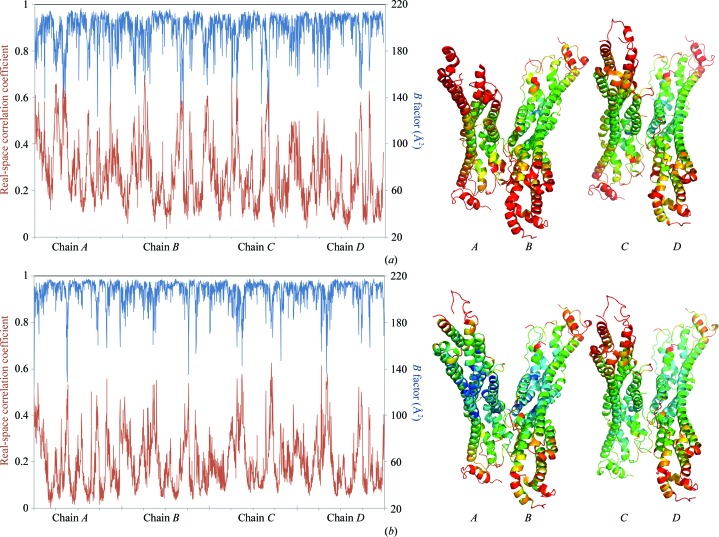
Plot of average *B* factor (blue) and real-space correlation coefficient (red) as a function of residue sequence number of the main-chain atoms of the *A*, *B*, *C* and *D* chains of (*a*) PDB entry 4acr and (*b*) the optimally dehydrated Gpc-1 structure. The cartoons of the asymmetric units are coloured by *B*-factor distribution, where dark blue = 10 Å^2^ and red = 100 Å^2^.

An extremely useful tool to gain an overview of model quality and to compare different models is by inspecting the plot of real-space correlation coefficients (RSCCs), which show how well the model fits the density map, on a residue-by-residue basis. Weak correlation indicates a poor electron-density fit, indicating a genuine absence of ordered regions or building errors (Brändén & Jones, 1990 ▶). The RSCC is often correlated to the refined atomic *B* factor. Fig. 7 ▶ shows a plot of the main-chain *B* factor and RSCC *versus* residue number for the control structure 4acr and the dehydrated model 4bwe. Chains *B* and *D* in 4acr show normal behaviour (Fig. 7 ▶ *a*), with the exception of three loop regions (L1, L2 and L3). Chain *C* shows a worrisome correlation between extremely poor real-space correlation and excessive *B* factors for the locally misbehaving protease-site lobe, which is completely invisible in chain *A* (70 missing residues). The plot also shows excessive *B* factors and poor RSCC for the whole of chain *A*. Fig. 7 ▶(*b*) shows the same plot for the model after dehydration, which shows similar and normal behaviour for all chains, with the exception of the three loops. After dehydration the overall model *B* factor fell from 73.8 to 61.4 Å^2^, which is in better agreement with the corresponding values found in 700 structures refined at the same resolution (using *phenix.polygon*). The average RSCC improved from 0.863 for 4acr to 0.901 for the dehydrated structure.

**Figure 8:**
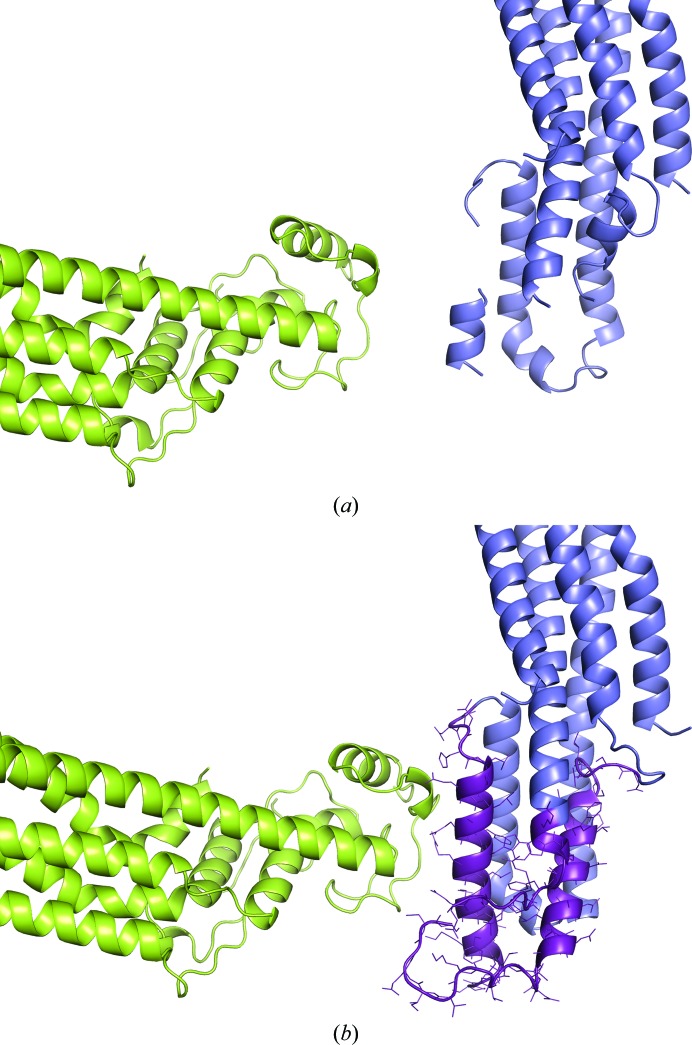
Crystal packing of chains *A* (slate blue) and *C* (lime green) of control (*a*) and dehydrated (*b*) models. The 60 residues of the protease-site lobe (violet) in chain *A* were ordered after dehydration.

*B* factors measure the atomic displacement of an atom from its mean position, which quantitates the uncertainty in the latter for each atom. Disorder may be owing to static variation in the atomic position in different unit cells, thermal vibration about equilibrium positions or dynamic effects of group movement (Rupp, 2010 ▶). During crystal dehydration new crystal contacts may form when the molecules rearrange and pack together more closely, which may reduce the disorder of some badly ordered regions inside the crystals. In the dehydrated Gpc-1 crystals the orientation with respect to each other of the pairs of chains *A*/*B* and *C*/*D* is preserved (almost no rotation and <1 Å translation of *B* relative to *A*), while the pair *C*/*D* undergoes an almost pure translation of about 11 Å relative to *A*/*B*. This translation results in the creation of a new intermolecular interface. In 4acr the protease-site lobe was disordered in chain *A*. After dehydration, this region (60 residues) became ordered, forming two helices and one loop (Fig. 8 ▶). This is owing to the creation of new lattice contacts between chain *A* and the copy of chain *C* related by the symmetry operation −*x* + 1, *y* − ½, −*z* + 1, with a total interface area of 375.3 Å^2^, owing to the translation of chains *C*/*D* relative to chains *A*/*B*.

Glypican-1 crystals, in space group *P*2_1_, exhibit translational pseudosymmetry. The native Patterson function for 4acr shows a peak at (½, 0.0193, ½) at 30% of the intensity of the origin peak (data not shown). This translation relates the monomer pairs *A*/*B* and *C*/*D*. Dehydration appears to increase the degree of pseudosymmetry in the structure. The trans­lation peak, now at (½, 0.0742, ½), increases to 42% of the height of the origin peak. Together with the reduction in the β angle from 94.6 to 90.8°, this suggests an ongoing transition to face-­centred orthorhombic symmetry, but within the range of parameters investigated here we did not achieve a complete transition.

## 4. Conclusion   

In this work, we have shown glypican-1 to be a successful case for improvement in diffraction properties by controlled crystal dehydration using the HC1 device. Through investigation of the optimal final humidity, the dehydration rate and the total incubation time, we achieved significant and reproducible improvements in crystal isotropy that led to an improved model for glypican-1. Although it is very difficult to deconvolute the effects of dehydration rate and incubation time, as they are correlated to a certain extent, we have demonstrated that the total incubation time is at least a factor to be reckoned with. In particular, incubation times that are too extended turned out to be detrimental to crystal quality. It is most likely that the success of dehydration is partly owing to the elongated nature of the molecule and the poor crystal packing in one direction. The method might be generally useful for glypicans, which are all predicted to have the same elongated structure by sequence homology and are likely to make deficient crystal contacts in at least one direction.

## Supplementary Material

PDB reference: glypican-1, 4bwe

## Figures and Tables

**Table 1:** Effect of total incubation time on glypican-1 crystal diffraction quality The four dehydration protocols resulting in the data with lowest anisotropy are highlighted in bold.

*T*_inc_ (min)	% RH	Dehydration rate (s per 0.1% RH step)	*a* ()	*b* ()	*c* ()	()	Unit-cell volume (^3^)	Solvent content (%)	Resolution ()	Mosaic spread ()	Anisotropy *B* (^2^)	Isotropic *B* factor (^2^)
0	95	0	47.17	168.63	147.8	94.6	1171553	54.9	2.6	0.26	40.7	46.0
27	88	6	47.16	166.96	137.7	90.5	1083719	51.3	3.1	0.24	24.3	44.8
50	91	60	46.92	166.40	136.7	90.4	1067488	50.6	2.8	0.27	26.1	48.6
55	90	40	47.00	166.50	137.5	90.5	1075652	50.9	2.7	0.15	21.5	40.6
**60**	**87**	**30**	**47.11**	**166.44**	**137.9**	**90.8**	**1081018**	**51.2**	**2.5**	**0.18**	**17.5**	**35.0**
**65**	**86**	**30**	**47.14**	**166.53**	**138.6**	**90.8**	**1087859**	**51.4**	**2.46**	**0.24**	**13.6**	**36.8**
**80**	**89**	**60**	**46.94**	**166.95**	**139.1**	**90.1**	**1090230**	**51.6**	**2.5**	**0.36**	**18.7**	**32.2**
**81**	**86**	**40**	**47.14**	**166.48**	**137.6**	**90.8**	**1079537**	**51.1**	**2.5**	**0.27**	**17.9**	**40.5**
85	86	30	47.15	166.44	138.4	90.9	1086221	51.4	2.7	0.23	28.4	45.0
93	87	60	46.86	165.82	136.1	90.5	1057342	50.1	2.6	0.47	22.5	44.2
98	86	50	46.95	165.71	134.2	90.2	1044234	49.5	3.5	0.45	60.4	71.1
105	86	60	46.47	163.24	124.7	86.6	944466	44.1	3.6	0.56	71.2	94.8

**Table 2:** Data-collection and refinement statistics Values in parentheses are for the highest resolution shell.

	4acr	Dehydrated model (4bwe)
Wavelength ()	1.0397	1.0000
Resolution range ()	29.72.55 (2.642.55)	29.52.46 (2.552.46)
Space group	*P*2_1_	*P*2_1_
Unit-cell parameters (, )	*a* = 47.2, *b* = 168.6, *c* = 147.8, = 90, = 94.6, = 90	*a* = 47.2, *b* = 166.7, *c* = 139.1, = 90, = 90.8, = 90
Total reflections	346094 (32013)	194774 (19151)
Unique reflections	74604 (7464)	75752 (7492)
Multiplicity	4.6 (4.3)	2.6 (2.6)
Completeness (%)	99.8 (99.2)	97.3 (97.3)
Mean *I*/(*I*)	12.4 (3.3)	7.6 (2.1)
Wilson *B* factor (^2^)	46.2	36.8
*R*_merge_(*I*)	0.088 (0.678)	0.098 (0.656)
*R*_model_(*F*)	0.251 (0.323)	0.231 (0.317)
*R*_free_(*F*)	0.292 (0.357)	0.278 (0.373)
No. of atoms		
Total	12812	13328
In macromolecules	12486	12887
In ligands	84	65
Waters	242	376
No. of protein residues	1592	1648
R.m.s.d., bonds ()	0.003	0.003
R.m.s.d., angles ()	0.72	0.76
Ramachandran favoured (%)	98	98
Ramachandran outliers (%)	0.13	0
Average *B* factor (^2^)	73.8	61.7
